# Respiratory tract infection and risk of bleeding in oral anticoagulant users: self-controlled case series

**DOI:** 10.1136/bmj-2021-068037

**Published:** 2021-12-21

**Authors:** Haroon Ahmed, Heather Whitaker, Daniel Farewell, Julia Hippisley-Cox, Simon Noble

**Affiliations:** 1 Division of Population Medicine, Cardiff University School of Medicine, Cardiff, UK; 2 Data and Analytical Sciences, Public Health England, London, UK; 3 Nuffield Department of Primary Care Health Sciences, University of Oxford, Oxford, UK

## Abstract

**Objective:**

To estimate the association between untreated, community acquired, respiratory tract infections and bleeding in oral anticoagulant users.

**Design:**

Self-controlled case series.

**Setting:**

General practices in England contributing data to the Clinical Practice Research Datalink GOLD.

**Participants:**

1208 adult users of warfarin or direct oral anticoagulants with a general practice or hospital admission record of a bleeding event between January 2010 and December 2019, and a general practice record of a consultation for a community acquired respiratory tract infection for which immediate antibiotics were not prescribed (that is, untreated).

**Main outcome measures:**

Relative incidence of major bleeding and clinically relevant non-major bleeding in the 0-14 days after an untreated respiratory tract infection, compared to unexposed time periods.

**Results:**

Of 1208 study participants, 58% (n=701) were male, median age at time of first bleed was 79 years (interquartile range 72-85), with a median observation period of 2.4 years (interquartile range 1.3-3.8). 292 major bleeds occurred during unexposed time periods and 41 in the 0-14 days after consultation for a respiratory tract infection. 1003 clinically relevant non-major bleeds occurred during unexposed time periods and 81 in the 0-14 days after consultation for a respiratory tract infection. After adjustment for age, season, and calendar year, the relative incidence of major bleeding (incidence rate ratio 2.68, 95% confidence interval 1.83 to 3.93) and clinically relevant non-major bleeding (2.32, 1.82 to 2.94) increased in the 0-14 days after an untreated respiratory tract infection. Findings were robust to several sensitivity analyses and did not differ by sex or type of oral anticoagulant.

**Conclusions:**

This study observed a greater than twofold increase in the risk of bleeding during the 0-14 days after an untreated respiratory tract infection. These findings have potential implications for how patients and clinicians manage oral anticoagulant use during an acute intercurrent illness and warrant further investigation into the potential risks and how they might be mitigated.

## Introduction

Most oral anticoagulants are initiated to prevent or treat venous thromboembolism, or to prevent stroke in people with atrial fibrillation.^1^ The main oral anticoagulants in use are warfarin and direct oral anticoagulants. Recent trends suggest that warfarin use has decreased but use of direct oral anticoagulants has increased substantially, leading to an overall increase in oral anticoagulant use of 54% in the United States and 71% in the United Kingdom.^2^ In the highest prescribing regions of England in 2019, prescription rates of warfarin and direct oral anticoagulants were 170 and 231 per 1000 residents, respectively.^3^ Although warfarin and direct oral anticoagulants can effectively prevent and treat thromboembolic events, they are also associated with morbidity and mortality from gastrointestinal and intracranial bleeding.^4^

Drug-drug interactions between warfarin and certain antibiotics, such as macrolides and fluoroquinolones, are well known causes of serious bleeding.^5^ ^6^ ^7^ Direct oral anticoagulants have fewer known drug-drug interactions than warfarin and have a more predictable anticoagulant response, resulting in fewer reports of antibiotic related harm. However, serious bleeding events are still reported, particularly after co-prescription of direct oral anticoagulants and macrolide antibiotics.^8^ ^9^ For warfarin and direct oral anticoagulants, it is unclear whether bleeding arises primarily from antibiotic-anticoagulant interactions, or whether attributable risk is from the underlying infection itself. The relation between severe infection and coagulopathy is well recognised^10^ but little evidence currently supports an association between milder community acquired infections and bleeding.

A small case-control study observed an association between excessive anticoagulation (confirmed by an international normalised ratio >7) in 31 warfarin users and a recent intercurrent illness.^11^ A recent retrospective cohort study estimated the risk of excessive anticoagulation in people with community acquired infection who received antibiotic treatment (antibiotic group) and those who did not receive antibiotic treatment (unwell controls), as well as controls without infection (stable controls).^12^ The proportion of people with a follow-up international normalised ratio of 5.0 or more were 3.2%, 2.6%, and 1.2% for the antibiotic group, unwell controls, and stable controls, respectively. Risk of an international normalised ratio of 5.0 or more was greater among the antibiotic and unwell control groups than among the stable control group. Hospital admission for any bleed was infrequent (67 bleeds in a cohort of 12 006 people) and similar across the three groups. Overall, the findings suggested bleeding risk was similar in people with community acquired infection irrespective of whether antibiotics were prescribed. However, the study might have been underpowered to detect a difference between the antibiotic group (n=5857) and unwell control group (n=570), and unmeasured differences between the two groups could have biased the findings towards the null. For example, a greater proportion of people in the unwell control group might have used over-the-counter treatments for symptom relief that elevated their international normalised ratios or increased the risk of bleeding, such as paracetamol^13^ or non-steroidal anti-inflammatory drugs (NSAIDs).^14^

Evidence of an association between community acquired infections and major bleeding could lead to targeted monitoring of international normalised ratios (for people prescribed warfarin) and pre-emptive dose change or other guidance for intercurrent illness (for users of any oral anticoagulant) to prevent some of these events. There are also implications for antibiotic prescribing, because currently the bleeding risk is thought to arise from antibiotic-anticoagulant interactions, but some of this risk could be attributable to the underlying infection. Therefore, this study aimed to estimate the association between community acquired respiratory tract infections (RTI) without immediate antibiotic prescription and a range of bleeding events. We used a self-controlled study design to reduce the impact of time invariant confounding between people.

## Methods

### Data source

We used anonymised longitudinal general practice data from the GOLD version of the UK Clinical Practice Research Datalink (CPRD).^15^ General practice records in the UK are a reliable source of data on disease diagnoses^16^ (recorded using Read and SNOMED codes), and drug prescriptions (recorded by use of codes from the Dictionary of Medicines and Devices). Practices contributing to CPRD GOLD are audited to assess the reliability and accuracy of data recording.^15^ Patient level data are also assessed and considered acceptable for inclusion in the CPRD if internally consistent in recording of age, sex, registration details, and clinical events. As of April 2021, CPRD GOLD contained data for 3.2 million living patients, with data deemed acceptable for research, registered at 408 practices across the UK that use Vision software to manage electronic health records.^17^ The CPRD GOLD sample represents 4.7% of the UK population and 4.6% of UK general practices.^15^ CPRD GOLD data were compared with 2011 UK census data and found to be broadly representative of the wider UK population in terms of age and sex distribution.^18^

Practices opt in to contribute data to CPRD, and about 50% provide additional consent to allow CPRD to link data at the patient level with other datasets, including hospital admission data.^19^ Previous studies have found that the characteristics of patients from practices with linked data were representative of the entire CPRD GOLD population in terms of age, sex, and deprivation.^20^ Bleeding events assessed and diagnosed in hospital are poorly recorded in primary care records^21^ and therefore, major bleeding outcomes in this study were ascertained from ICD-10 (international classification of diseases, 10th revision) codes recorded in linked hospital admission data.

### Study design

We carried out a self-controlled case series study in which individuals acted as their own controls.^22^ Comparisons are made within individuals rather than between individuals as in a cohort or case-control study. Only those people who have experienced both a bleeding event of interest (outcome) and an untreated RTI (exposure) are included. Self-controlled case series investigate the effect of a time varying exposure on an outcome by comparing the incidence of outcome events within periods of prespecified excess risk from the exposure with the incidence of outcome events during all other times.^23^ In this study, we were interested in excess risk during the 0-90 days after a general practice consultation for an RTI for which immediate antibiotics were not prescribed. We divided this 90 day risk period into smaller risk windows. The 0-14 day risk window was our prespecified primary risk window of interest, but we also investigated risk windows of 15-30, 30-60, and 60-90 days. A period of seven days before the general practice consultation for RTI was included as a pre-risk period. Time outside of the risk and pre-risk periods was regarded as unexposed time—that is, time not related to risk from a general practice consultation for untreated RTI.

The temporal association between an exposure and an outcome is estimated by Poisson models to derive incidence rate ratios, comparing the rate of the outcome during an individual’s exposed and unexposed times.^24^ Self-controlled case series have been applied in various settings, including to investigate common infective exposures,^25^ ^26^ and are particularly useful when an appropriately comparable control group would be difficult to identify. Further details about the study design and its assumptions are described in eAppendix 1.

### Population and follow-up

The source population were 4 553 515 people who contributed at least one day of data to CPRD GOLD between 1 January 2011 and 31 December 2019, and whose data were deemed acceptable for research, and eligible for linkage to hospital admission data. From the source population, we identified people who had their first ever prescription of warfarin or a direct oral anticoagulant within the study period of 1 January 2011 to 31 December 2019. For inclusion, the date of the first prescription needed to be after the year of their 18th birthday, and after the date when their practice’s data were regarded as up to standard.

The observation period began on the date of the first new prescription of warfarin or a direct oral anticoagulant, and ended on the earliest of three dates: end of the treatment period (of warfarin or a direct oral anticoagulant), death, end of CPRD data collection, or end of the study period (31 December 2019). The end of the treatment period was defined as the earliest of two dates: 90 days after the date of the last prescription of the drug that was initiated, or the date of the first prescription for a different oral anticoagulant. Thus, the observation period only included the treatment period of a person’s first ever oral anticoagulant, akin to a new user incident design in a cohort study.^27^

### Outcomes

For inclusion in the self-controlled case series analysis, an individual needed to have experienced an outcome and exposure of interest within their observation period. The primary outcome was informed by the definition of major bleeding by the subcommittee on control of anticoagulation of the Scientific and Standardization Committee (SSC) of the International Society on Thrombosis and Haemostasis (ISTH). The committee defines major bleeds as those that result in death, are life threatening, cause chronic sequelae, or consume major healthcare resources.^28^ For this study, we defined major bleeding as a hospital admission for intracerebral or gastrointestinal bleeding. These events are commonly encountered major bleeds and have been ascertained from UK health records by many studies, increasing confidence in the reliability and completeness of their recording in routine health data.^4^ ^21^ ^29^ These bleeds reflected both a pragmatic approach to ascertainment of major bleeding and acknowledgment of the ISTH criteria for major bleeding. 

Major bleeding was ascertained from ICD-10 codes recorded in linked hospital admission data and included codes recorded at any point during a hospital stay and in any position within the hierarchy of diagnoses for a hospital admission. The secondary outcomes were events indicating a less severe bleed. The outcome definition was adapted from the ISTH SSC’s criteria for clinically relevant non-major bleeding (CRNMB)^30^ of a bleed that did not fit the criteria of major bleeding but that required medical intervention, hospital admission, or face-to-face evaluation. For this study, we defined CRNMB as a general practice consultation or hospital admission for haemoptysis, epistaxis, or haematuria, which we ascertained from general practice and hospital admission data using a combination of Read and ICD-10 codes. Code lists are available in eAppendix 2.

### Exposure and risk periods

The exposure in this study was a combination of a general practice consultation for an RTI without immediate antibiotic prescription, which we refer to as an untreated RTI but acknowledge that some people could have used prescribed or over-the-counter non-antibiotic treatments such as cold remedies or NSAIDs. Exposure was ascertained from Read codes that represented possible symptoms or diagnoses of upper RTIs (including inner ear, nose, and throat infections), lower RTIs, and influenza. We did not include Read codes with a high likelihood of representing a clinical presentation that would require immediate antibiotics (eg, bacterial pneumonia). The frequency of recorded Read codes for the RTIs included in the final self-controlled case series are available in eFigures 1 and 2.

An RTI might start several days before a general practice consultation and serious bleeding during this time could be misclassified as occurring during the unexposed period. Therefore, we included a pre-risk period that started seven days before the date of the RTI consultation and ended on the date of the RTI consultation.

The risk period started on the date of the RTI consultation and ended on the earliest of five dates: 90 days after the date of the RTI consultation; date of an antibiotic prescription (because we were interested in the risk during the untreated period only); death; end of CPRD data collection; and the end of study period. 

### Multiple exposures and outcomes

**Fig 1:**
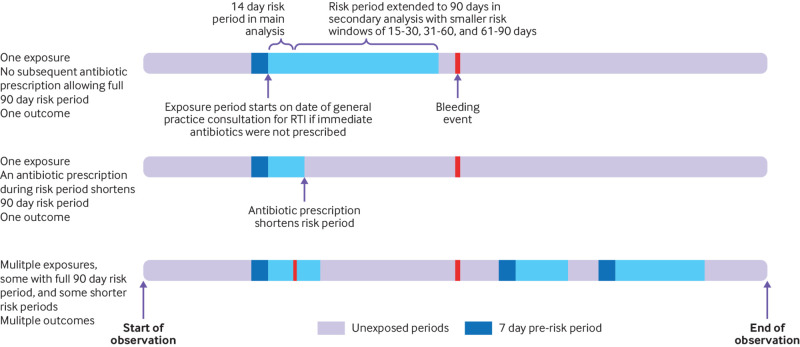
Study design of self-controlled case series, with examples of possible combinations of exposures and outcomes. RTI=respiratory tract infection

For exposures and outcomes, we regarded Read codes occurring within 28 days of each other as relating to the same event. A 28 day clear period was required before a code was regarded as relating to a new exposure or outcome. We included multiple exposures and multiple outcomes. Time outside of the period lasting from the start of a seven day pre-risk period to the end of the 90 day risk period was regarded as unexposed time. Figure 1 shows a graphical representation of the self-controlled case series with examples of the possible combinations of study exposures and outcomes.

### Covariates

The self-controlled case series design implicitly controls for confounders that remain constant over time, such as sex. We adjusted for three time varying confounders: age with 40 age bands, using quantiles of age at first outcome to define each band; year to adjust for changes in health behaviour and clinical management over the study period that could influence how exposures and outcomes were recorded; and season, defined as winter (December-February), spring (March-May), summer (June-August), and autumn (September-November) to reflect the seasonal incidence of RTIs.

### Statistical analyses

We used descriptive statistics to characterise the sample of patients included in the self-controlled case series analysis. We calculated the number of events and person time for the pre-risk, risk, and unexposed periods. We used conditional Poisson regression to estimate incidence rate ratios and 95% confidence intervals for the relative incidence of bleeding events during exposed versus unexposed periods, adjusted for age, calendar time, and season. We also fitted a spline based age effect, where the relative age effect was represented by a smooth function obtained by splicing together polynomials of low dimension, to ensure that we had adjusted flexibly for age effects.^31^

In prespecified sensitivity analyses, we considered alternative pre-risk periods of three days, and five days. We also excluded people who died within four weeks of an event to explore bias arising from a bleeding event affecting the length and timing of the observation period (violation of the self-controlled study assumptions).^32^ We did several post hoc sensitivity analyses. We explored the impact of subdividing the 14 day risk window into smaller periods. We explored whether the association between RTI and bleeding differed if we restricted to the first exposure or outcome, and if we included all RTIs irrespective of whether they received immediate antibiotic treatment or not. The effect modification by sex or type of oral anticoagulant was also explored. The main analysis was repeated using ocular and external ear infections as a negative control exposure, because these infections induce a mild and more localised inflammatory response than RTIs. For people using warfarin, we looked for codes suggestive of monitoring or dose change during the 14 day risk window to explore whether that might affect our findings. Finally, in response to peer review, we explored whether NSAIDs prescribed on the day of the RTI might confound the association between RTI and bleeding. Data management and analyses were carried out in RStudio. Self-controlled case series models were fitted using the self-controlled case series package.^33^

### Patient and public involvement

Patients from the lead author’s (HA) practice who used warfarin or direct oral anticoagulants were involved from the design phase of this study and helped shape the question and objectives. Three formal patient representatives were recruited from Health and Care Research Wales and Anticoagulation UK during the design phase and provided valuable support with interpretation of study findings, particularly around how oral anticoagulant users self-manage their drug treatment during intercurrent illness.

## Results

**Fig 2:**
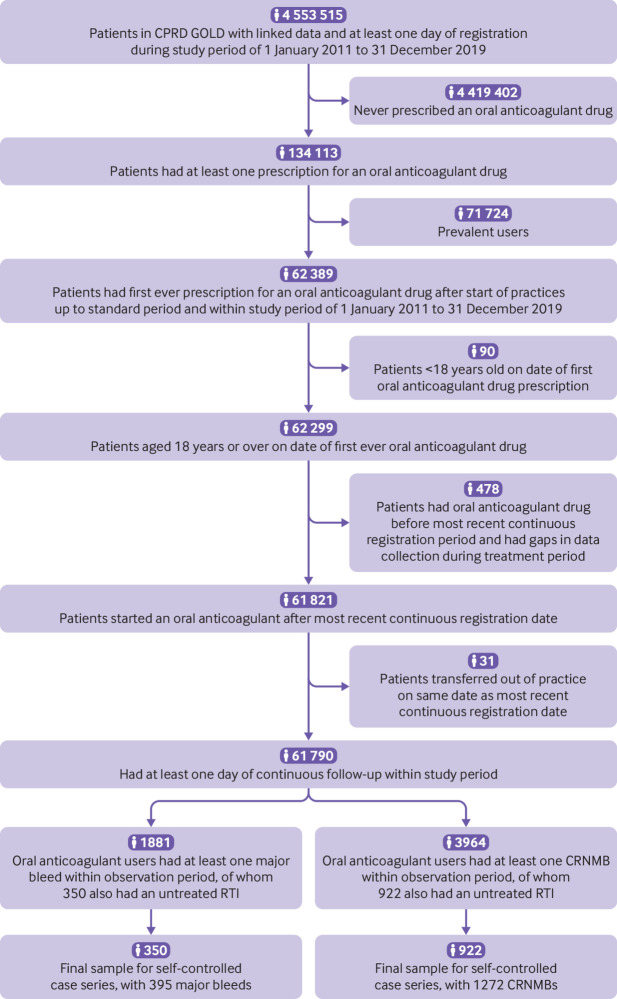
Flow of patients into analyses of self-controlled case series. CPRD=Clinical Practice Research Datalink; RTI=respiratory tract infection; CRNMB=clinically relevant non-major bleeding

Of 61 790 eligible incident users of oral anticoagulants, 1109 warfarin users and 772 users of direct oral anticoagulants had at least one major bleed, and 2538 warfarin users and 1426 users of direct oral anticoagulants had at least one CRNMB, within their observation period. Of the 5845 oral anticoagulant users who had at least one bleed, 1208 had at least one untreated RTI within their observation period and comprised the sample for the self-controlled case series analysis. This sample included 350 people who had 395 major bleeds, and 922 people who had 1272 CRNMBs (64 people had both a major bleed and a CRNMB; fig 2). Of 1208 adult participants, 58% (n=701) were male, median number of years of observation were 2.4 (interquartile range 1.3-3.8), median age at initiation of incident oral anticoagulation was 77 years (70-83), and median age at time of first bleed was 79 years (72-85). The most common major bleed was gastrointestinal bleeding (336/395, 85%), and the most common CRNMB was haematuria (587/1272, 46%; table 1). The 1208 people included in the self-controlled case series had similar characteristics to those 60 582 people who were not included in terms of age, deprivation, smoking, alcohol intake, and choice of anticoagulant, but had longer treatment periods (eTable 1).

**Table 1:** Characteristics of 1208 adults who had at least one bleeding event and at least one untreated respiratory tract infection during the observation period. Data are number (%) of participants unless otherwise stated

Characteristic	No (%) of participants
Sex:	
Male	701 (58)
Female	507 (42)
Start of observation period:	
2011-12	435 (36)
2013-14	384 (32)
2015-16	231 (19)
2017-18	142 (12)
2019	16 (1)
Median (IQR) No of years of observation	2.4 (1.3-3.8)
Median (IQR) age (years) at start of observation period	77 (70-83)
Index of multiple deprivation at start of observation period*:	
1 (least deprived)	252 (21)
2	285 (24)
3	274 (23)
4	222 (18)
5 (most deprived)	175 (14)
Most recently recorded alcohol intake at start of observation period:	
Current drinker	486 (40)
Ex-drinker	49 (4)
Non-drinker	254 (21)
Missing	419 (35)
Most recently recorded smoking status at start of observation period:	
Current smoker	118 (10)
Ex-smoker	528 (44)
Non-smoker	497 (41)
Missing	65 (5)
First oral anticoagulant prescribed during the observation period:	
Apixaban	144 (12)
Dabigatran	26 (2)
Edoxaban	<5 (<1)
Rivaroxaban	229 (19)
Warfarin	806 (67)
Major bleeds:	
Intracranial bleed	59 (15)
Gastrointestinal bleed	336 (85)
Clinically relevant non-major bleeds:	
Haemoptysis	212 (17)
Epistaxis	473 (37)
Haematuria	587 (46)
Median (IQR) age (years) on date of first event	79 (72-85)
No of respiratory tract infections per person:	
1	878 (73)
2	229 (19)
3	63 (5)
4	28 (2)
≥5	10 (1)

IQR=interquartile range.

* Divided by quintile.

### Major bleeding

We identified 395 major bleeds, of which 292 occurred during 287 579 days of observation of the unexposed period and 41 occurred during 6710 days of observation of the 0-14 day risk window. We observed an increase in the relative incidence of major bleeding in the 0-14 days after participants had an untreated RTI (incidence rate ratio 2.70, 95% confidence interval 1.85 to 3.94; table 2). The incidence rate ratio remained relatively unchanged when we adjusted for the confounding effects of age, calendar year, and season (2.68, 1.83 to 3.93), and when we modelled age as a spline based function (2.65, 1.76 to 4.00). We did not observe a statistically significant increase in the relative incidence of major bleeding during the 15-30, 31-60, or 61-90 day risk windows.

**Table 2:** Incidence rate ratios (IRR) for major bleeding and clinically relevant non-major bleeding (CRNMB) in people who had at least one untreated respiratory tract infection during the observation period

Time period	No of events	Total No of days of observation	Crude IRR (95% CI)	Age adjusted IRR (95% CI)	Age, season, and year (adjusted IRR (95% CI))
Major bleeding:					
Baseline	292	287 579	1	1	1
Pre-risk*	4	3332	0.60 (0.22 to 1.63)	0.60 (0.22 to 1.63)	0.62 (0.22 to 1.63)
0-14 days	41	6710	2.70 (1.85 to 3.94)	2.68 (1.83 to 3.92)	2.68 (1.83 to 3.93)
15-30 days	12	5899	0.64 (0.28 to 1.46)	0.62 (0.27 to 1.42)	0.62 (0.27 to 1.42)
31-60 days	22	9667	0.76 (0.41 to 1.41)	0.72 (0.38 to 1.34)	0.72 (0.38 to 1.35)
61-90 days	24	7825	1.41 (0.82 to 2.43)	1.34 (0.77 to 2.31)	1.35 (0.78 to 2.34)
CRNMB:					
Baseline	1003	827 042	1	1	1
Pre-risk*	17	9114	1.00 (0.61 to 1.62)	1.00 (0.61 to 1.61)	0.99 (0.61 to 1.60)
0-14 days	81	23 166	2.33 (1.83 to 2.96)	2.33 (1.84 to 2.97)	2.32 (1.82 to 2.94)
15-30 days	49	21 149	1.39 (0.99 to 1.94)	1.39 (0.99 to 1.96)	1.38 (0.98 to 1.94)
31-60 days	66	34 767	1.09 (0.80 to 1.48)	1.09 (0.80 to 1.49)	1.08 (0.79 to 1.47)
61-90 days	56	28 188	0.91 (0.62 to 1.33)	0.91 (0.63 to 1.34)	0.90 (0.62 to 1.32)

* Pre-risk refers to the seven day period before a general practice consultation for a respiratory tract infection.

### Clinically relevant non-major bleeding

Of 1272 CRNMBs identified, 1003 occurred during 827 042 days of observation of the unexposed period and 81 occurred during 23 166 days of observation of the 0-14 day risk window. We observed an increase in the relative incidence of CRNMB in the 0-14 days after participants had an untreated RTI (incidence rate ratio 2.33, 95% confidence interval 1.83 to 2.96; table 2). The incidence rate ratio remained relatively unchanged when we adjusted for the confounding effects of age, calendar year, and season (2.32, 1.82 to 2.94), and when we modelled age as a spline based function (2.09, 1.60 to 2.76). As with major bleeding, we did not observe a statistically significant increase in the relative incidence of CRNMB during the 15-30, 31-60, or 61-90 day risk windows.

### Sensitivity analyses

Changing the pre-risk period to three days or five days made no substantial difference to the risk estimates. When identifying deaths within the observation period, we found that 124/350 people (35%) died after a major bleed (median time to death 94 days, interquartile range 16.8-449.5), and 316/922 (34%) died after a CRNMB (441 days, 153.8-966.8). We repeated the main analyses excluding 38 people who died within 28 days of a major bleed and 16 people who died within 28 days of a CRNMB, but this adjustment made no difference to the risk estimates (eTable 2). We divided the 14 day risk window into five-day risk windows and found that relative incidence peaked in the 11-15 day window for major bleeding (incidence rate ratio 3.04, 95% confidence interval 1.63 to 5.65) and the 0-5 day window for CRNMB (3.94, 2.93 to 5.30; eTable 3). Restricting the analysis to people who only had one RTI or restricting to only the first bleed outcome made little difference to the risk estimates (eTables 4 and 5). 

We repeated the analyses with all RTIs, irrespective of whether patients received immediate antibiotic treatment, and included 1258 RTIs for major bleeding (770 (61%) treated) and 3482 RTIs for CRNMB (2154 (62%) treated). For those participants receiving immediate antibiotics, the most common were amoxicillin (67%), doxycycline (15%), and clarithromycin (8%). We observed a small reduction in the magnitude of the relative incidence of major bleeding (incidence rate ratio 2.28, 95% confidence interval 1.74 to 3.00) and CRNMB (2.17, 1.85 to 2.54), compared with our main analysis (table 3). We found no evidence of effect modification by sex (eTable 6) or by type of anticoagulant used (table 4). No significant associations were observed between eye and external ear infections and major bleeding or CRNMB (major bleeding 0.74, 0.30 to 1.83; CRNMB 1.26, 0.84 to 1.89; eTable 7). We identified a Read code suggestive of warfarin monitoring or dose change within the 14 day risk period after 88 (26%) RTIs in people who had a major bleed, and 301 (23%) RTIs in those people who had a CRNMB. Few RTIs were associated with an NSAID prescription on the day of consultation (n=0 in people with a major bleed, n=3 in those with a CRNMB) or at any time in the 90 days before the consultation (n=24, n=37) suggesting that prescribed NSAID use was not likely to be an important temporal confounder.

**Table 3:** Incidence rate ratios (IRR) for major bleeding and clinically relevant non-major bleeding for analysis including participants with any RTI, irrespective of whether immediate antibiotics were prescribed or not

Time period	No of events	Total No of days of observation	Age, season, and year (adjusted IRR (95% CI))
Major bleeding:			
Baseline	466	429 801	1
Pre-risk*	7	8659	0.49 (0.23 to 1.05)
0-14 days	68	19 348	2.28 (1.74 to 3.00)
15-30 days	39	20 239	1.34 (0.94 to 1.90)
31-60 days	66	36 900	1.27 (0.96 to 1.68)
61-90 days	58	31 946	1.20 (0.88 to 1.63)
Clinically relevant non-major bleeding:			
Baseline	1584	1 211 799	1
Pre-risk*	42	24 115	1.01 (0.74 to 1.37)
0-14 days	192	66 922	2.17 (1.85 to 2.54)
15-30 days	105	70 376	1.17 (0.95 to 1.45)
31-60 days	163	129 400	1.02 (0.85 to 1.21)
61-90 days	162	113 418	1.02 (0.85 to 1.23)

* Pre-risk refers to the seven day period before a general practice consultation for a respiratory tract infection.

**Table 4:** Incidence rate ratios (IRR) for major bleeding and clinically relevant non-major bleeding (CRNMB) according to type of anticoagulant used by participants

Time period	Major bleeding					CRNMB			
	No of events	Total No of days of observation	Age, season, and calendar year (adjusted IRR (95% CI))	P value for interaction*		No of events	Total No of days of observation	Age, season, and calendar year (adjusted IRR (95% CI))	P value for interaction*
Warfarin:				P=0.57					P=0.96
Baseline	168	185 924	1			685	598 198	1	
Pre-risk†	2	2128	0.50 (0.12 to 2.03)			13	6468	1.10 (0.63 to 1.92)	
0-14 days	26	4317	2.95 (1.83 to 4.75)			55	16 513	2.30 (1.72 to 3.07)	
15-30 days	9	3811	0.70 (0.25 to 1.94)			28	15 140	1.28 (0.84 to 1.95)	
31-60 days	18	6087	1.04 (0.51 to 2.11)			49	25 278	1.10 (0.76 to 1.60)	
61-90 days	14	4871	1.23 (0.58 to 2.61)			40	20 622	0.89 (0.56 to 1.40)	
Direct oral anticoagulants:									
Baseline	124	101 655	1			318	228 844	1	
Pre-risk†	2	1204	0.74 (0.18 to 3.03)			4	2646	0.70 (0.26 to 1.90)	
0-14 days	15	2393	2.20 (1.15 to 4.23)			26	6653	2.21 (1.44 to 3.40)	
15-30 days	3	2088	0.50 (0.12 to 2.07)			21	6009	1.52 (0.86 to 2.70)	
31-60 days	4	3580	0.30 (0.07 to 1.27)			17	9489	0.98 (0.55 to 1.74)	
61-90 days	10	2954	1.48 (0.65 to 3.37)			16	7566	0.87 (0.44 to 1.72)	

* Calculated by the likelihood ratio test.

† Pre-risk refers to the seven day period before a general practice consultation for a respiratory tract infection.

## Discussion

### Principal findings

In this self-controlled case series study of 1208 oral anticoagulant users, we observed a greater than twofold increase in the risk of major bleeding and CRNMB in the 0-14 days after an untreated RTI. The direction and magnitude of the association remained relatively unchanged across several sensitivity analyses and did not appear to be modified by sex or type of oral anticoagulant. Exploratory analyses suggest that the risk peaks occur at 0-5 days for CRNMB and 11-15 days for major bleeding. We observed a small reduction in the magnitude of the association when we included all RTIs, irrespective of whether patients received immediate antibiotic treatment or not.

### Strengths and limitations of this study

We used a self-controlled case series design to investigate the association between untreated RTI and bleeding in oral anticoagulant users, adjusting for key time varying confounders: age, year, and season. The study design implicitly accounted for time invariant confounders by comparing exposed and unexposed periods within the same person. Several sensitivity analyses assessed the robustness of our findings. We used recommended approaches to resolve potential violations of the self-controlled case series method assumptions, including the use of a pre-risk period, and restricting the analysis to the first major bleeding or CRNMB only. Long term treatments such as oral anticoagulants are well recorded in UK general practice, facilitating reliable identification of the population of interest and the period of observation. We used linked hospital data to maximise capture of bleeding events. Previous work found that only 20% of bleeding events recorded in hospital data had a corresponding code in general practice data, and the use of linked data enabled capture of a greater proportion of relevant outcomes.^21^

This study had several limitations. Participants having an RTI were ascertained from general practice records and represented an RTI for which the patient consulted a healthcare professional, and which led to a corresponding record in their health data. This does not reflect all community acquired RTIs because population based surveys suggest that only around 20% of people in England with recent RTI symptoms consulted their general practitioner, and those who consulted were concerned that their symptoms were more severe or had lasted longer than expected.^34^ This approach also does not capture RTIs seen in out-of-hours general practice or other forms of urgent primary care. 

The exposure was an RTI without immediate antibiotic prescription and some people not prescribed immediate antibiotics could have acquired them from elsewhere. However, the impact of this limitation is likely to be negligible given the lack of availability of antibiotics in the UK without a prescription, and given the findings from previous research that the proportion of people with RTI symptoms who use non-prescribed antibiotics might be as low as 0.4%.^34^ Furthermore, the inclusion of all RTIs into the analysis, irrespective of whether patients received immediate antibiotic treatment or not, only had a small impact on the estimated incidence rate ratios. Although we referred to the exposure in this study design as untreated RTI, some participants could have sought other treatments (eg, NSAIDs) for their symptoms, which might be a source of residual confounding. We explored the use of prescribed NSAIDs but were unable to account for over-the-counter drugs, which could be important given that survey data from 1000 adults in England with a recent RTI indicated that 60% of these people sought over-the-counter treatments.^35^ However, NSAIDs are a well recognised cause of bleeding in oral anticoagulant users and use could be minimal, as reflected by the low prevalence of prescribed NSAID use in our study sample.

Despite the large source population, our final sample was smaller than expected for the study analyses, and the study could have been underpowered to detect significant associations beyond our 14 day risk window. Our observation period was based on oral anticoagulant prescriptions, not anticoagulant use, and we were unable to further estimate adherence or the impact of non-adherence. Our findings could have been affected by clinicians pre-emptively advising additional monitoring or reduced dosing for people taking warfarin who consulted with an RTI. We identified Read codes suggesting that additional monitoring or reduced dosing might have been advised in roughly a quarter of untreated RTIs in our sample of warfarin users. Our patient representative also highlighted that people who use warfarin and self-monitor might, like herself, monitor more frequently during an intercurrent illness. However, the combined effect of these factors (increased monitoring, early action for changes in coagulation) is likely to reduce the risk of bleeding and thus bias risk estimates towards the null.

### Comparison with other studies

Few studies have investigated the impact of acute infection on bleeding risk in oral anticoagulant users, but several studies have investigated the impact of acute infection on the international normalised ratio in warfarin users. Recent intercurrent illness was reported four times more frequently in warfarin users with an elevated international normalised ratio in a case-control study (31 cases, 100 controls).^11^ Warfarin users in another case-control study (67 cases, 81 controls) more frequently reported a recent infection (adjusted odds ratio 1.76).^35^ Both studies had wide confidence intervals, indicating considerable uncertainty around the point estimates and reflecting the small sample size. Our study also observed an association between acute infection and coagulation related adverse outcomes; however, our study was considerably larger than these other studies, had more precise estimates, and included in the study design an exposure that was ascertained from clinical records rather than recall. Furthermore, our outcome of bleeding is clinically more relevant than an elevated international normalised ratio.

Further evidence for a relation between acute infection and elevated international normalised ratios is presented in a retrospective cohort study that analysed data from Kaiser Permanente Colorado’s integrated healthcare delivery system.^12^ Warfarin users who consulted for an RTI but did not purchase an antibiotic (untreated) were more likely to have a follow-up international normalised ratio of ≥5 than stable controls without an RTI. Patients with a treated RTI were also more likely to have a follow-up international normalised ratio of ≥5.0 than stable controls, but not more likely than those people with an untreated RTI. These findings accord with ours in the present study—of an association between untreated RTI and bleeding, and of only a small reduction in the magnitude of this association when we included treated and untreated RTIs in the analysis. However, our study adds to the limited understanding of this area through its use of a range of well defined bleeding outcomes and its investigation of risk in people using direct oral anticoagulants. Potential reasons why untreated RTI might increase the risk of excessive anticoagulation and a subsequent bleed include the effect of interactions with over-the-counter cough and cold treatments containing paracetamol,^36^ ^37^ and increased clotting factor catabolism secondary to fever.^38^

### Policy implications and conclusions

These findings are important and timely given the increasing rates of oral anticoagulant use and the lack of knowledge and guidance on how to manage oral anticoagulant use during acute infection. We were unable to find specific details of how infection might affect anticoagulation in clinical guidelines. The National Institute for Health and Care Excellence’s clinical knowledge summary was recently amended and now states that “acute illness may exaggerate the effect of warfarin and necessitate a dose reduction,” but no similar statement is provided for direct oral anticoagulants.^39^ Insights from our patient representatives suggest that some patients who use warfarin suspect that acute infection could affect their coagulation and change their monitoring and other behaviours during intercurrent illness. However, we were unable to find any published research to support this.

Future studies should replicate this work with a larger sample, which will help assess whether we were underpowered to detect a difference beyond the 14 day risk window. Studies of the association between antibiotic use and bleeding have observed effects of up to 60 days after exposure.^5^ If these findings are confirmed in further studies, qualitative research is needed to understand knowledge and behaviours of patients and clinicians on managing anticoagulant use during acute infection. Research should subsequently inform co-production of guidance to help mitigate any additional risk and reduce the frequency of bleeding related adverse outcomes.

This self-controlled case series observed a greater than twofold increase in the risk of major bleeding and CRNMB in the 0-14 days after an untreated RTI, with some suggestion that the risk peaks at 0-5 days for CRNMB and 11-15 days for major bleeding. This finding has potential implications for how patients and clinicians manage oral anticoagulant use during an acute intercurrent illness, but further work is needed before any clinical recommendations are made. However, these findings warrant further investigation to fully understand the potential risks and how they might be mitigated.

**What is already known on this topic**

One previous observational study found that the risk of an elevated international normalised ratio was similar between people with respiratory tract infection, irrespective of whether they received antibiotics
Evidence of an association between community acquired infections and major bleeding has implications for anticoagulant management and antibiotic prescribing
A self-controlled study design was used to investigate this association, to reduce the impact of time invariant confounding between people

**What this study adds**

In this self-controlled case series of 1208 oral anticoagulant users, a greater than twofold increase was seen in the risk of major bleeding and clinically relevant non-major bleeding in the 0-14 days after a respiratory tract infection for which no antibiotics were prescribed
The direction and magnitude of the association remained relatively unchanged across several sensitivity analyses and did not appear to be modified by sex or type of oral anticoagulant
These findings are important and timely given the increasing rates of oral anticoagulant use and the lack of knowledge and guidance on how to manage oral anticoagulant use during acute infection

## Data Availability

The Clinical Practice Research Datalink data agreement prevents data sharing but researchers can apply directly to the Clinical Practice Research Datalink for a dataset.

## Web extra

Extra material supplied by authors

Web appendix: Supplementary material

## Notes

The lead author (HA) affirms that this manuscript is an honest, accurate, and transparent account of the study being reported; that no important aspects of the study have been omitted; and that any discrepancies from the study as planned have been explained.

Dissemination to participants and related patient and public communities: We will share our results with the public through a press release issued by Cardiff University and taking part in media interviews where possible. We will also work with our patient and public representatives to write a lay summary for dissemination to organisations of patients using oral anticoagulants, and summarise key findings and messages from this research for dissemination on social media including the relevant Twitter feeds.

Provenance and peer review: Not commissioned; externally peer reviewed.
