# Supplementary material for: Respiratory tract infection and risk of bleeding in oral anticoagulant users: self-controlled case series
Source: BMJ. 2021 Dec 21;375:e068037. doi: 10.1136/bmj-2021-068037 (PMC8689396; doi:10.1136/bmj-2021-068037)
Supplement: Supplementary file 1 — Web appendix: Supplementary material [file ahmh068037.ww.pdf]

Respiratory tract infection and risk of bleeding for oral anticoagulant medication users: self-controlled case series

Supplementary material

e-appendix 1.

The self-controlled case series (SCCS) method and assumptions

The main assumptions of the SCCS method are:

1.Recurrent outcomes must be independent. We included recurrent outcomes in this study's main analysis. Recurrent major bleeds were rare (39/350, 11%) but recurrent Clinically Relevant Non-Major Bleeds (CRNMBs) were relatively common (245/922, 27%). Several factors that may increase a patient's susceptibility to a bleed may persist after a first bleed, e.g., co-morbidities, alcohol intake, response to anticoagulant dosing, and therefore a first bleed may influence the chance of subsequent bleeds. If this assumption is violated, a valid approach to overcome this issue is to consider only the first incident bleed.[1,2] We repeated the main analysis but restricted to only the first bleed and found no appreciable difference in the relative incidence estimates (e-table 4).
2.The occurrence of an outcome should not alter the probability of subsequent exposure. A major bleed may reduce the probability of a subsequent community acquired respiratory tract infection (exposure) due to the potential for prolonged hospital stay or death, but also may increase the probability of a subsequent community acquired respiratory tract infection if it causes impairments to respiratory function. In the latter case, bleeds would therefore be more likely to occur in the immediate pre-exposure period and thus lead to a falsely inflated baseline incidence and an underestimate of the relative incidence during the risk period. A valid approach to overcome this issue is to label a predefined time-period prior to exposure as a "pre-risk" period, that is not included in the unexposed (baseline) period.[3] We therefore included a 7-day pre-risk period in this study.
3.The outcome must not censor the observation period. Major bleeds can potentially lead to death and censor the observation period. Therefore, we repeated the main analysis but excluded people who died within four weeks of an event to explore bias arising from a bleeding event affecting the length and timing of the observation period. found no appreciable difference in the relative incidence estimates (e-table 1).

References

[1] Whitaker HJ, Farrington CP, Spiessens B, et al. Tutorial in biostatistics: the self-controlled case series method. Stat Med 2006;25(10):1768-97. doi: 10.1002/sim.2302

[2] Farrington CP, Hocine MN. Within-individual dependence in self-controlled case series models for recurrent events. Journal of the Royal Statistical Society: Series C (Applied Statistics) 2010;59(3):457-75. doi: 10.1111/j.1467-9876.2009.00703.x

[3] Whitaker HJ, Ghebremichael-Weldeselassie Y, Douglas IJ, Smeeth L, Farrington CP. Investigating the assumptions if the self-controlled case series method. Stat Med 2018; 37(4):643-658. doi: 10.1002/sim.7536

e-appendix 2.

Code lists for major bleeding and clinically relevant non-major bleeding (CRNMB)

**Major bleeding**	**ICD-10 codes**	**CPRD GOLD medcodes**
Intracranial bleeding	I60, I60.0, I60.1, I60.2, I60.3, I60.4, I60.5, I60.6, I60.7, I60.8, I60.9, I61, I61.0, I61.1, I61.2, I61.3, I61.4, I61.5, I61.6, I61.7, I61.8, I61.9, I62, I62.0, I62.1, I62.9, I69.0, I69.1, I69.2	N/A
Upper gastrointestinal bleeding	I85, I85.0, K29.0, K92.0, K92.1, K92.2, K25.0, K25.2, K25.4, K25.6, K26.0, K26.2, K26.4, K26.6, K27.0, K27.2, K27.4, K27.6, K28.0, K28.2, K28.4, K28.6	N/A
Lower gastrointestinal bleeding	K62.5	N/A
**CRNMB**	**ICD-10 codes**	**CPRD GOLD medcodes**
Epistaxis	R04, R04.0, R04.1	501, 5785, 99744, 4594, 5382, 51571, 5793, 99524, 4594, 15540
Haematuria	R31	507, 6247, 6659, 7232, 6030, 9651, 17060, 20357, 7164, 107296, 6901, 47228, 6234
Haemoptysis	R04.2, R04.8, R04.9	4135, 2244, 107548, 33742, 1610,10013

e-figure 1. Frequency of codes used for RTIs in people with major bleeding.

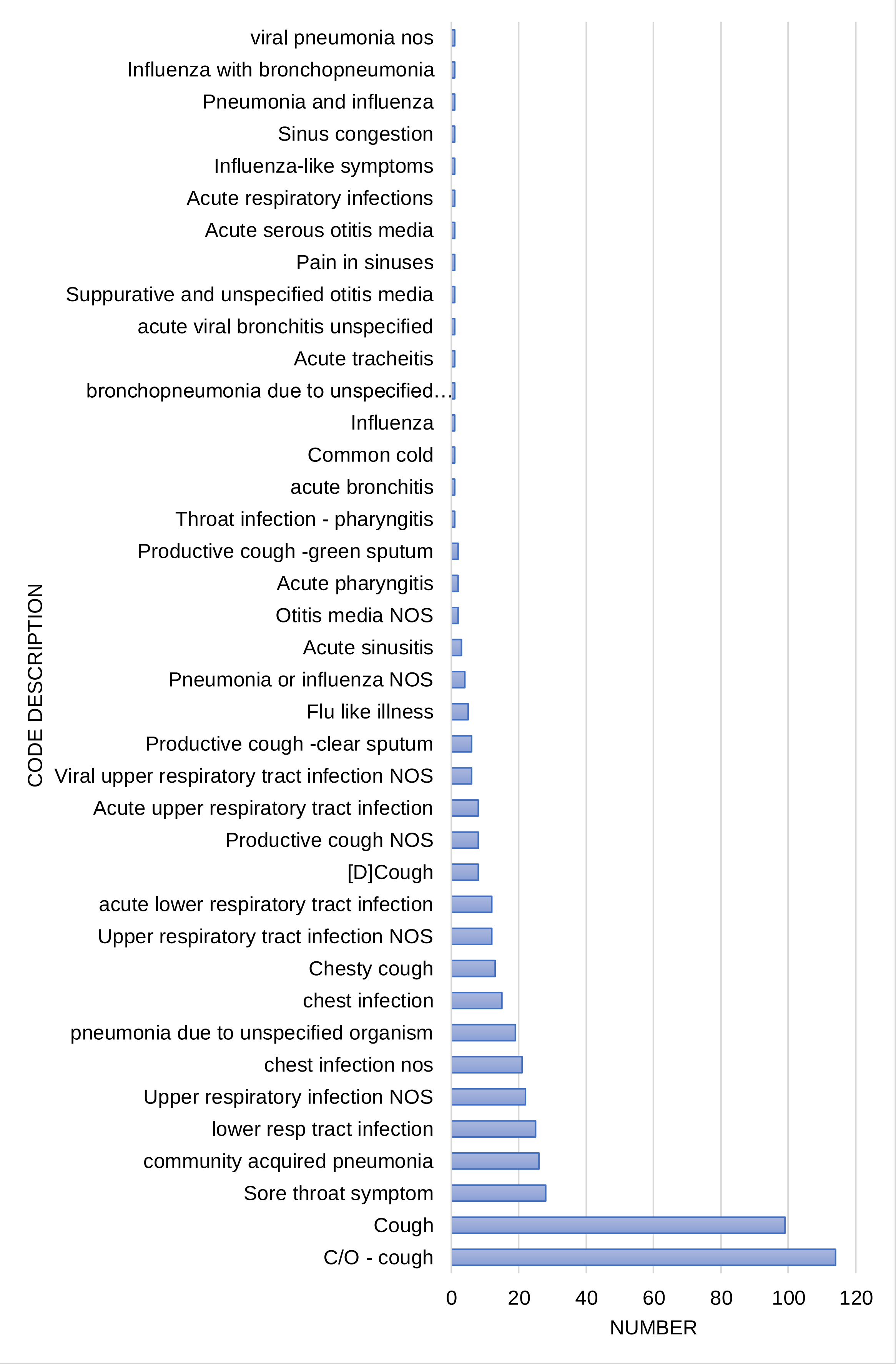

e-figure 2. Frequency of codes used for RTIs in people with clinically relevant non-major bleeding.

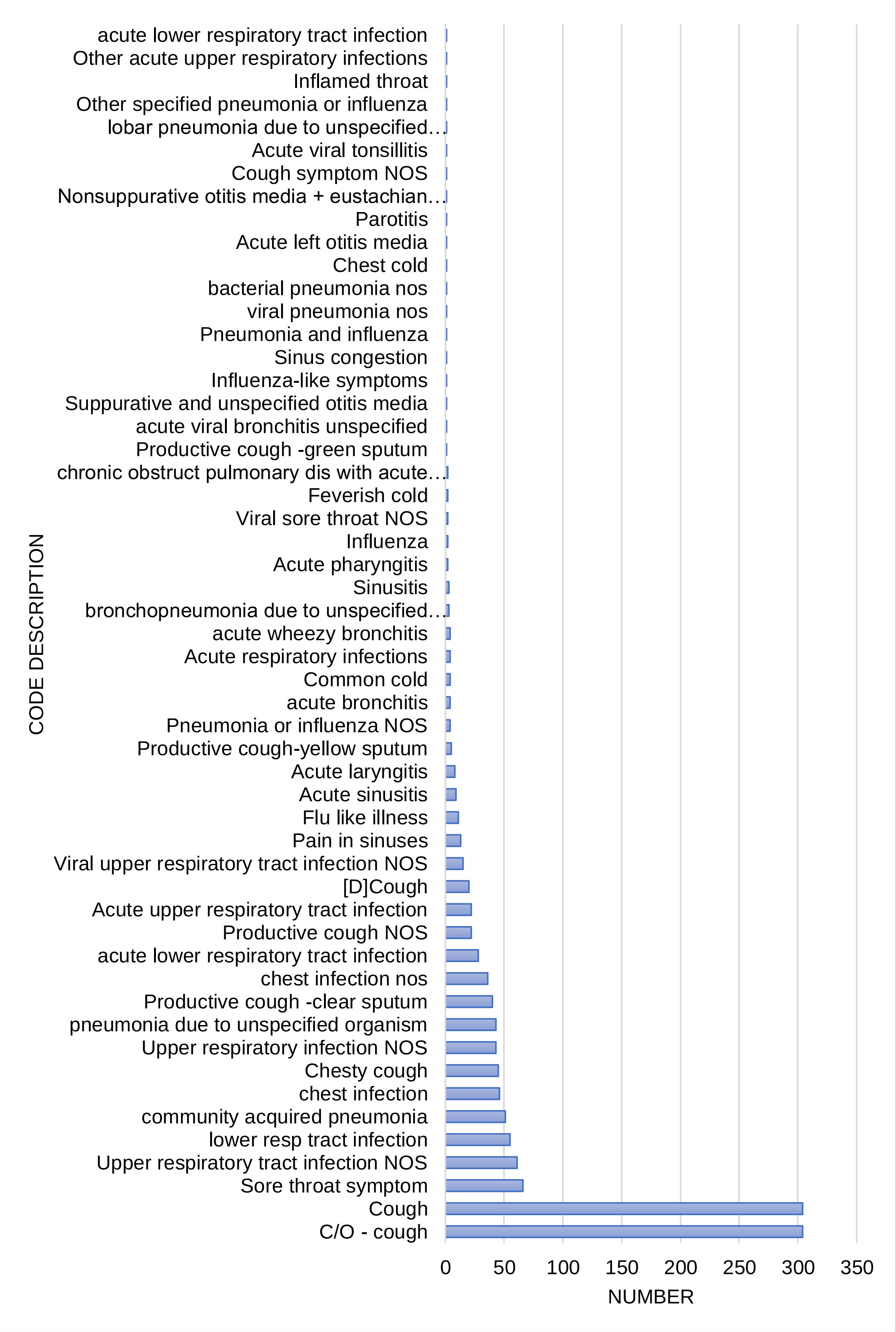

e-table 1. Characteristics of 1208 oral anticoagulant users included in the SCCS (had a relevant event and exposure) compared to 60,582 oral anticoagulant users not included in the SCCS. Values are numbers (%) unless otherwise stated.

Characteristic	1208 adults included in the SCCS	60,582 adults not included in the SCCS
Sex:		
Male	701 (58)	32117 (53)
Female	507 (42)	28465 (47)
Start of observation period:		
2011-2012	435 (36)	16751 (28)
2013-2014	384 (32)	17730 (29)
2015-2016	231 (19)	13970 (23)
2017-2018	142 (12)	8638 (14)
2019	16 (1)	3493 (6)
Median (IQR) years of observation	2.4 (1.3-3.8)	0.66 (0.3-1.5)
Median (IQR) age in years at start of observation period	77 (70-83)	74 (64-82)
Index of multiple deprivation quintile at start of observation period:		
1 (least deprived)	252 (21)	15316 (25)
2	285 (24)	14420 (24)
3	274 (23)	12277 (20)
4	222 (18)	10516 (17)
5 (most deprived)	175 (14)	8031 (13)
Missing	0	22 (<1)
Most recent recorded alcohol intake at start of observation period:		
Current drinker	486 (40)	24085 (40)
Ex-drinker	49 (4)	2629 (4)
Non-drinker	254 (21)	12198 (20)
Missing	419 (35)	21670 (36)
Most recent recorded smoking status at start of observation period:		
Current smoker	118 (10)	7494 (12)
Ex-smoker	528 (44)	20879 (34)
Non-smoker	497 (41)	29408 (49)
Missing	65 (5)	2801 (5)
First oral anticoagulant prescribed during the observation period:		
Apixaban	144 (12)	8628 (14)
Dabigatran	26 (2)	1771 (3)
Edoxaban	<5 (<1)	698 (1)
Rivaroxaban	229 (19)	13134 (22)
Warfarin	806 (67)	36351 (60)

e-table 2. Incidence rate ratios (IRR) for major bleeding and clinically relevant non-major bleeding (CRNMB) excluding people who died within four weeks of a bleed.

	Major bleeding number of events = 357	CRNMB number of events = 1256
Time period	Age, season, and calendar year adjusted IRR (95% CI)	Age, season, and calendar year adjusted IRR (95% CI)
Baseline	1	1
Pre-risk	0.73 (0.27-1.98)	1.03 (0.63-1.67)
0-14	2.70 (1.79-4.06)	2.30 (1.80-2.93)
15-30	0.68 (0.30-1.55)	1.37 (0.97-1.93)
31-60	0.42 (0.18-0.96)	1.07 (0.78-1.47)
61-90	1.34 (0.76-2.36)	0.91 (0.63-1.33)

e-table 3. Incidence rate ratios (IRR) for major bleeding and clinically relevant non-major bleeding (CRNMB) with the 14-day risk period subdivided into smaller 5-day windows

	Major bleeding number of events = 395	CRNMB number of events = 1272
Time period	Age, season, and calendar year adjusted IRR (95% CI)	Age, season, and calendar year adjusted IRR (95% CI)
Baseline	1	1
0-5 days	2.81 (1.59-4.97)	3.94 (2.93-5.30)
6-10 days	2.42 (1.27-4.62)	1.44 (0.88-2.37)
11-15 days	3.04 (1.63-5.65)	1.29 (0.74-2.24)
16-20 days	0.60 (0.15-2.45)	1.08 (0.58-2.02)
20-90 days	0.93 (0.62-1.40)	1.09 (0.88-1.36)

e-table 4. Incidence rate ratios (IRR) for major bleeding and clinically relevant non-major bleeding (CRNMB) restricting to people who only had one exposure period.

	Major bleeding number of events = 302	CRNMB number of events = 883
Time period	Age, season, and calendar year adjusted IRR (95% CI)	Age, season, and calendar year adjusted IRR (95% CI)
Baseline	1	1
Pre-risk	0.60 (0.19-1.88)	0.92 (0.49-1.73)
0-14	2.71 (1.74-4.24)	2.43 (1.81-3.27)
15-30	0.27 (0.07-1.11)	0.99 (0.60-1.64)
31-60	0.83 (0.43-1.63)	1.03 (0.69-1.54)
61-90	1.30 (0.68-2.49)	0.95 (0.59-1.51)

e-table 5. Incidence rate ratios (IRR) for major bleeding and clinically relevant non-major bleeding (CRNMB) restricting to only the first outcome.

	Major bleeding number of events = 350	CRNMB number of events = 922
Time period	Age, season, and calendar year adjusted IRR (95% CI)	Age, season, and calendar year adjusted IRR (95% CI)
Baseline	1	1
Pre-risk	0.67 (0.25-1.82)	0.97 (0.56-1.69)
0-14	2.92 (1.98-4.32)	2.51 (1.93-3.28)
15-30	0.70 (0.31-1.61)	1.30 (0.87-1.94)
31-60	0.74 (0.38-1.43)	1.03 (0.71-1.48)
61-90	1.46 (0.83-2.58)	0.79 (0.50-1.27)

e-table 6. Incidence rate ratios (IRR) for major bleeding and clinically relevant non-major bleeding (CRNMB) by sex.

	Major bleeding		CRNMB	
Time period	Age, season, and calendar year adjusted IRR (95% CI)	P value for interaction (calculated using likelihood ratio test)	Age, season, and calendar year adjusted IRR (95% CI)	P value for interaction (calculated using likelihood ratio test)
Sex				
Men:		p=0.693		p=0.850
Baseline	1		1	
Pre-risk	0.93 (0.29-2.95)		1.00 (0.54-1.81)	
0-14	2.42 (1.38-4.26)		2.36 (1.76-3.18)	
15-30	0.40 (0.10-1.64)		1.30 (0.85-1.99)	
31-60	0.91 (0.41-2.03)		1.01 (0.69-1.49)	
61-90	1.33 (0.60-3.00)		0.75 (0.46-1.23)	
Women:				
Baseline	1		1	
Pre-risk	0.28 (0.04-2.02)		0.99 (0.44-2.24)	
0-14	2.80 (1.64-4.77)		2.24 (1.49-3.38)	
15-30	0.84 (0.30-2.35)		1.55 (0.88-2.73)	
31-60	0.52 (0.19-1.46)		1.24 (0.73-2.12)	
61-90	1.34 (0.63-2.87)		1.27 (0.70-2.28)	

e-table 7. Incidence rate ratios (IRR) for major bleeding and clinically relevant non-major bleeding (CRNMB) using eye and external ear infections as a negative control exposure.

	Major bleeding<br>number of events = 224	CRNMB<br>number of events = 759
Time period	Age, season, and calendar year adjusted IRR (95% CI)	Age, season, and calendar year adjusted IRR (95% CI)
Baseline	1	1
Pre-risk	no events	0.62 (0.28-1.40)
0-14	0.74 (0.30-1.83)	1.26 (0.84-1.89)
15-30	1.40 (0.70-2.80)	0.81 (0.49-1.33)
31-60	0.58 (0.27-1.27)	0.87 (0.60-1.27)
61-90	0.80 (0.40-1.60)	1.18 (0.84-1.65)
